# Impact of tight glucose control on circulating 3-hydroxybutyrate in critically ill patients

**DOI:** 10.1186/s13054-021-03772-6

**Published:** 2021-10-25

**Authors:** Jan Gunst, Astrid De Bruyn, Michael P. Casaer, Sarah Vander Perre, Lies Langouche, Greet Van den Berghe

**Affiliations:** Clinical Division and Laboratory of Intensive Care Medicine, Department of Cellular and Molecular Medicine, KU Leuven, 3000 Leuven, Belgium

**Keywords:** Tight glucose control, Hyperglycemia, Insulin, Critical illness, Ketogenesis, Ketone body, Early parenteral nutrition

## Abstract

**Background:**

Recent evidence suggests a potentially protective effect of increasing ketone body availability via accepting low macronutrient intake early after onset of critical illness. The impact of blood glucose control with insulin on circulating ketones is unclear. Whereas lowering blood glucose may activate ketogenesis, high insulin concentrations may have the opposite effect. We hypothesized that the previously reported protective effects of tight glucose control in critically ill patients receiving early parenteral nutrition may have been mediated in part by activation of ketogenesis.

**Methods:**

This is a secondary analysis of 3 randomized controlled trials on tight versus liberal blood glucose control in the intensive care unit, including 700 critically ill children and 2748 critically ill adults. All patients received early parenteral nutrition as part of the contemporary standard of care. Before studying a potential mediator role of circulating ketones in improving outcome, we performed a time course analysis to investigate whether tight glucose control significantly affected ketogenesis and to identify a day of maximal effect, if any. We quantified plasma/serum 3-hydroxybutyrate concentrations from intensive care unit admission until day 3 in 2 matched subsets of 100 critically ill children and 100 critically ill adults. Univariable differences between groups were investigated by Kruskal-Wallis test. Differences in 3-hydroxybutyrate concentrations between study days were investigated by Wilcoxon signed-rank test.

**Results:**

In critically ill children and adults receiving early parenteral nutrition, tight glucose control, as compared with liberal glucose control, lowered mean morning blood glucose on days 1–3 (*P* < 0.0001) via infusing insulin at a higher dose (*P* < 0.0001). Throughout the study period, caloric intake was not different between groups. In both children and adults, tight glucose control did not affect 3-hydroxybutyrate concentrations, which were suppressed on ICU days 1–3 and significantly lower than the ICU admission values for both groups (*P* < 0.0001).

**Conclusion:**

Tight versus liberal glucose control in the context of early parenteral nutrition did not affect 3-hydroxybutyrate concentrations in critically ill patients. Hence, the protective effects of tight glucose control in this context cannot be attributed to increased ketone body availability.

**Supplementary Information:**

The online version contains supplementary material available at 10.1186/s13054-021-03772-6.

## Introduction

Increasing evidence suggests a potentially protective effect of enhancing ketone availability during critical illness [1]. Whereas ketone bodies may serve as vital and more energy-efficient energy substrate than glucose or fatty acids, ketones may also exert signaling functions leading to anti-inflammatory effects and activation of recovery processes such as muscle regeneration and autophagy [2]. In line with this, studies performed in animal models of sepsis and brain injury have demonstrated protective effects of providing ketones or ketogenic diets [3, 4]. However, human evidence remains scarce, with only a few small randomized controlled trials (RCTs) suggesting improved blood glucose control by ketone supplementation or ketogenic diets [1].

Although ketogenesis has traditionally been reported to be blunted in critical illness [5–7], a recent pilot crossover RCT revealed that four hours of full fasting significantly increased blood ketone concentrations in long-stay critically ill patients [8]. Moreover, we recently demonstrated that withholding parenteral nutrition during the first week of intensive care unit (ICU) stay and hereby temporarily accepting insufficient enteral nutrition, significantly increased ketogenesis in critically ill children and adults [9, 10]. Activation of ketogenesis by such virtual fasting early during critical illness was most pronounced in children, in whom increased ketone availability also statistically mediated part of the outcome benefit of the intervention [9].

A second metabolic intervention that may affect ketone availability during critical illness is tight glucose control with insulin therapy [6, 11], although the net impact of the intervention on ketone concentrations has not been well documented. Indeed, although insulin is a known suppressor of ketogenesis [11–13], critical illness is characterized by profound insulin resistance in the liver–﻿a major site of ketone production–﻿﻿﻿, and in contrast to peripheral insulin resistance, hepatic insulin resistance is difficult to overcome with insulin therapy [14]. Moreover, elevated glucose concentrations may suppress lipolysis and subsequent ketogenesis, whereas lowering blood glucose concentrations could reduce suppression of ketogenesis [6, 11]. In this regard, a pilot crossover RCT in critically ill adults found activated ketogenesis by lowering blood glucose with insulin therapy [15]. However, in this crossover study, patients received significantly less feeding in periods on tight glucose control, which may have confounded the results. Hence, these findings require confirmation by larger RCTs with equal nutritional intake in both groups.

Our research group previously demonstrated that lowering blood glucose concentrations to the healthy, age-adjusted fasting range significantly improved morbidity and mortality of critically ill children and adults receiving early parenteral nutrition, as compared to tolerating stress hyperglycemia [16–18]. We hypothesize that part of the outcome benefit with tight glucose control in this context may have been mediated by increasing availability of ketone bodies through a mechanism primarily driven by lowering glucose concentrations in the setting of hepatic insulin resistance. We investigated this hypothesis in a secondary analysis of the original RCTs.

## Methods

### Patients and study design

This is a secondary analysis of 3 single-center RCTs performed in the adult surgical, adult medical (NCT00115479) and pediatric ICU (NCT00214916) in Leuven, Belgium, which demonstrated improved clinical outcome by tight glucose control in the ICU [16–18]. The study was performed in accordance with the 1964 Declaration of Helsinki and later amendments, and written informed consent was obtained from the patient or next of kin, or (for children) from the parents or legal guardian. The detailed study protocols and the effect on the primary outcome have been published [16–18]. In brief, patients were randomized upon ICU admission to target age-adjusted normal fasting blood glucose concentrations (50–80 mg/dL [2.8–4.4 mmol/L] for neonates and infants, 70–100 mg/dL [3.9–5.6 mmol/L] for children older than 1 year, 80–110 mg/dL [4.4–6.1 mmol/L] for adults) with insulin therapy (tight glucose control), or to liberal blood glucose control, whereby stress hyperglycemia was only treated when it exceeded 215 mg/dL (11.9 mmol/L). All patients received early parenteral nutrition to supplement insufficient or failing enteral nutrition as part of the contemporary standard of care. In all patients, blood samples were taken upon ICU admission, and thereafter daily at 6 ± 2 h am. In patients included in the adult medical ICU (*N* = 1200) and pediatric ICU study (*N* = 700), plasma and serum samples were collected. In the adult surgical ICU study (*N* = 1548), only serum samples were collected. After aliquoting, samples were stored at -80 °C until further analysis.

**Fig. 1:**
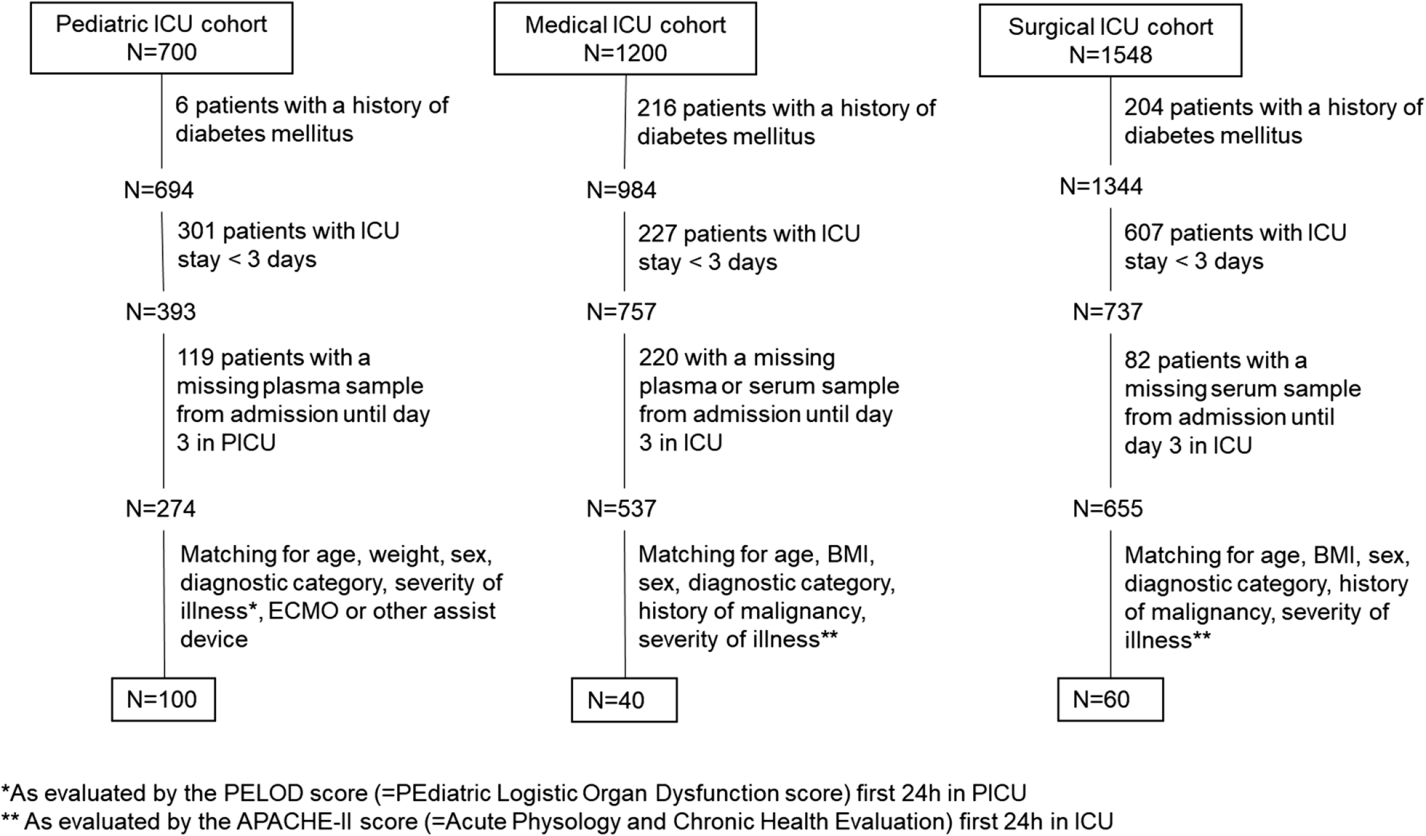
Selection and matching

In this secondary analysis, we aimed to study whether enhanced ketogenesis could have mediated part of the outcome benefit of tight glucose control seen in the parent RCTs [16–18]. Before studying such potential mediator role through multivariable regression analysis, we performed a time course analysis in 2 matched subsets of critically ill children and adults, to study whether tight glucose control significantly affected ketogenesis in these patients, and to identify a day of maximal effect (if any). To that purpose, we quantified daily circulating 3-hydroxybutyrate (3HB) concentrations from ICU admission until day 3 in the ICU. Patients were eligible if they required intensive care for least 3 days, did not have a history of diabetes mellitus and had available blood samples at each time point (upon admission and the 3 consecutive mornings). To rule out selection bias due to an impact of the intervention on ICU length of stay, and for feasibility reasons, the eligible pediatric (*N* = 274), adult medical (*N* = 537) and adult surgical (*N* = 655) patient cohorts were reduced with propensity score matching to obtain 2 matched cohorts of 100 adult and 100 pediatric patients (50 allocated to tight glucose control and 50 allocated to liberal glucose control, of whom (for the adult cohort) 40 from the medical ICU study, and 60 from the surgical ICU study, to obtain a proportional sample from the respective parent RCTs) (Fig. 1). The groups were matched for demographics (age and sex; weight of children, body mass index of adults), baseline type and severity of illness (Pediatric Logistic Organ Dysfunction (PELOD) score for children, Acute Physiology and Chronic Health Evaluation II (APACHE-II) score for adults), need of extracorporeal membrane oxygenation or mechanical hemodynamic support upon admission (for children) and history of malignancy (for adults).

### Laboratory analyses

Blood glucose was measured in whole blood by a blood gas analyzer (ABL700, Radiometer Medical, Copenhagen, Denmark). The concentration of 3HB was quantified with a laboratory assay based upon the oxidation of 3HB to acetoacetate by the enzyme 3HB dehydrogenase and the concomitant reduction of NAD^+^ to NADH, as previously described for plasma samples [9, 19]. Samples with undetectable 3HB concentrations were assigned the detection limit (0.04 mmol/L). In the pediatric cohort, 3HB was determined in plasma samples. As plasma was not available for the adult surgical ICU study, we first evaluated the validity of serum as a replacement for plasma by performing 3HB measurements in paired samples of adult patients included in the medical ICU study (40 patients with 4 paired samples, leading to 160 paired measurements). The mean difference of these paired measurements was 0.015 mmol/L (95% confidence interval ranging from -0.117 to 0.133 mmol/L). Since this confirmed validity of serum as replacement for plasma, 3HB was subsequently quantified in all serum samples of the adult cohort.

### Statistical analyses

Data are presented as frequencies and percentages or as medians with interquartile range. Fisher’s exact test and Kruskal-Wallis test were used to analyze univariable differences between patient groups, as appropriate. Wilcoxon signed-rank test was used to analyze paired differences in ketone concentrations between study days. All analyses were performed with the use of JMP software, version pro 15 (SAS Institute, NC, USA). Statistical significance was set at a *P* value of 0.05.

## Results

### Effect of tight versus liberal glucose control on plasma 3HB concentrations in critically ill children

**Fig. 2:**
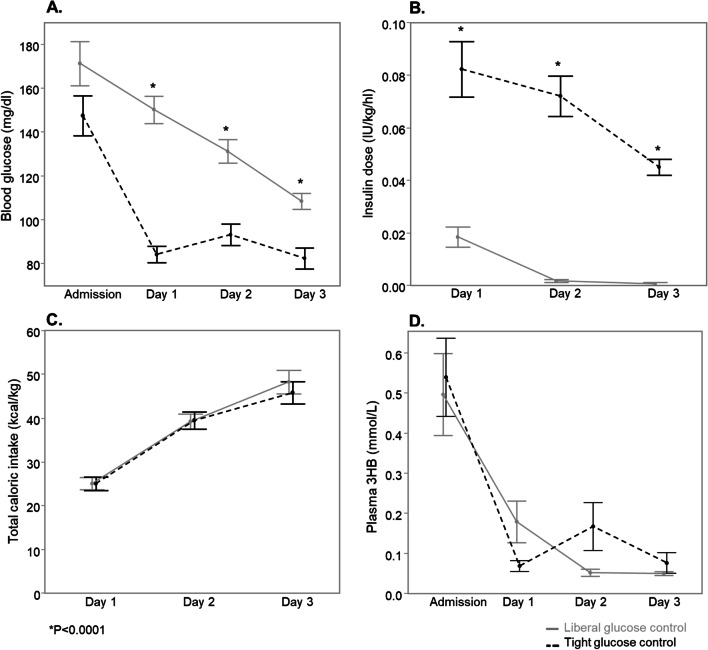
Admission and morning blood glucose concentrations (**a**), insulin dose (**b**), total caloric intake through enteral and parenteral nutrition (**c**) and plasma 3-hydroxybutyrate (3HB) concentrations (**d**) during the first 3 days in the ICU in a matched subset of critically ill children randomized to tight (dotted black line) or liberal (full gray line) glucose control. Data are shown as mean and standard error. Samples with undetectable 3HB concentrations were assigned the detection limit (0.04 mmol/L): 47 upon ICU admission (23 and 24 in tight and liberal group, respectively), 84 at day 1 (45 and 39 in tight and liberal group, respectively), 91 at day 2 (43 and 48 in tight and liberal group, respectively) and 92 at day 3 (46 in each group). * *P* ≤ 0.0001. For conversion of blood glucose in mg/dL to mmol/L, divide by 18

The matched cohort of critically ill children consisted of 50 patients randomized to tight glucose control and 50 patients randomized to liberal glucose control (Fig. 1). Baseline characteristics were similar between both groups (Table 1). Upon admission, blood glucose was comparable between both groups (*P* = 0.08), after which morning blood glucose was significantly lower in tight glucose control patients on all study days (all *P* < 0.0001) (Fig. 2a), in the presence of higher insulin doses (all *P* < 0.0001) (Fig. 2b) and similar intake through enteral and parenteral nutrition (Fig. 2c). From admission onward, plasma 3HB concentrations were similarly low in both randomization groups (Fig. 2d), with a significant decrease in concentrations between ICU admission and day 1 (*P* < 0.0001). Of all 400 3HB determinations, concomitant hypoglycemia (≤ 40 mg/dL [≤ 2.2 mmol/L]) on the corresponding blood glucose measurement was present at 5 occasions (1 blood glucose measurement upon randomization in each randomization group, thereafter 1 measurement at each time point in the tight glucose control group).

**Table 1:** Baseline characteristics and outcome of matched children

Baseline characteristics	Liberal GC (*N* = 50)	Tight GC (*N* = 50)	*P* value
Age (years)—median (IQR)	0.9 (0.2–2.9)	0.6 (0.2–3.1)	0.79
Age group^a^			0.39
Neonate—no. (%)	4 (8)	8 (16)	
Infant—no. (%)	23 (46)	19 (38)	
Child—no. (%)	23 (46)	23 (46)	
Weight (kg)—median (IQR)	7.7 (4.2–11.5)	6.6 (3.8–13.9)	0.75
Sex (male)—no. (%)	25 (50)	27 (54)	0.84
Diagnostic category			1.00
Surgical			
Transplantation—no. (%)	1 (2)	1 (2)	
Cardiac surgery—no. (%)	41 (82)	40 (80)	
Other surgery—no. (%)	4 (8)	5 (10)	
Medical—no. (%)	4 (8)	4 (8)	
PELOD first 24 h in ICU—median (IQR)	12 (11–12)	12 (11–12)	0.53
ECMO or other assist device—no. (%)	1 (2)	1 (2)	1.00

Outcome			
ICU mortality—no. (%)	4 (8)	1 (2)	0.36
Length of ICU stay (days)—median (IQR)	5 (3–9)	6 (3–10)	0.80
Patients with secondary infection—no. (%)	28 (56)	23 (46)	0.42

^a^Neonate corresponds to children aged < 4 weeks, Infants to children aged between 4 weeks and 1 year, Child to children aged > 1 year. BMI is body mass index, PELOD is Pediatric Logistic Organ Dysfunction score, ECMO is extracorporeal membrane oxygenation, GC is glucose control, IQR is interquartile range

### Effect of tight versus liberal glucose control on serum 3HB concentrations in critically ill adults

**Fig. 3:**
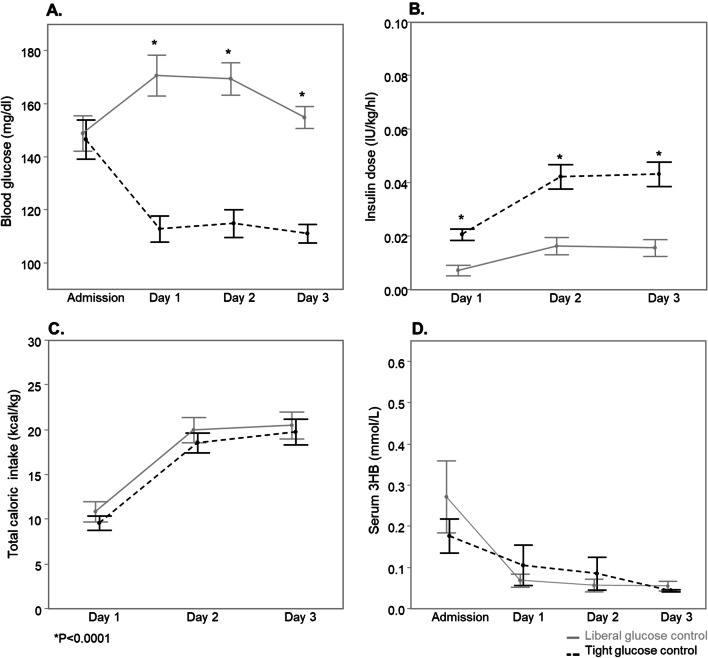
Admission and morning blood glucose concentrations (**a**), insulin dose (**b**), total caloric intake through enteral and parenteral nutrition (**c**) and serum 3-hydroxybutyrate (3HB) concentrations (**d**) during the first 3 days in the ICU in a matched subset of critically ill adults randomized to tight (dotted black line) or liberal (full gray line) glucose control. Data are shown as mean and standard error. Samples with undetectable 3HB concentrations were assigned the detection limit (0.04 mmol/L): 69 upon ICU admission (34 and 35 in tight and liberal group, respectively), 92 at day 1 (46 in each group), 94 at day 2 (46 and 48 in tight and liberal group, respectively) and 95 at day 3 (48 and 47 in tight and liberal group, respectively). *P ≤ 0.0001. For conversion of blood glucose in mg/dL to mmol/L, divide by 18

The matched cohort of critically ill adults consisted of 100 patients–50 patients per randomization group–﻿of whom 60 were admitted to the surgical ICU and 40 to the medical ICU (Fig. 1). Baseline characteristics were similar (Table 2, Additional file 1: Table S1). As was the case in the total adult study population [20], ICU mortality was significantly lower in tight glucose control patients (*P* = 0.01). Blood glucose concentrations were not different upon ICU admission (*P* = 0.6) and were significantly lower in tight glucose control patients throughout the first 3 days in ICU (all *P* < 0.0001) (Fig. 3a), through infusion of higher insulin doses (all *P* < 0.0001) (Fig. 3b) and in the presence of similar caloric intake (Fig. 3c). In both study groups, serum 3HB was similarly suppressed from days 1 to 3 (Fig. 3d), with a significant decrease from ICU admission to day 1 (*P* < 0.0001). Of all 400 3HB determinations, concomitant hypoglycemia was present on one corresponding blood glucose measurement at day 1 in a patient receiving liberal glucose control.

**Table 2:** Baseline characteristics and outcome of matched adults

Baseline characteristics	Liberal GC (*N* = 50)	Tight GC (*N* = 50)	*P* Value
Age (years)—median (IQR)	65 (56–74)	66 (55–74)	0.73
Length (cm)—median (IQR)	170 (165–176)	172 (170–179)	0.27
BMI (kg/m^2^)—median (IQR)	24.2 (21.6–27.7)	24.8 (22.0–27.5)	0.53
Sex (male)—no. (%)	41 (82)	45 (90)	0.39
Diagnostic group—no. (%)			0.93
Surgical			
Transplantation—no. (%)	4 (8)	2 (4)	
Cardiac surgery—no. (%)	17 (34)	19 (38)	
Other surgery—no. (%)	9 (18)	9 (18)	
Medical—no. (%)	20 (40)	20 (40)	
History of malignancy—no. (%)	6 (12)	6 (12)	1.00
APACHE-II score—median (IQR)	12 (8–19)	12 (8–21)	0.93

Outcome			
ICU mortality—no. (%)	16 (32)	5 (10)	0.01
Length of ICU stay (days)—median (IQR)	8 (4–18)	6 (4–14)	0.33

BMI is body mass index, APACHE-II is Acute Physiology and Chronic Health Evaluation II, IQR is interquartile range, GC is glucose control

## Discussion

In this secondary analysis of three RCTs on the impact of tight glucose control in critically ill children and adults receiving early parenteral nutrition, we found no impact of the intervention on circulating ketone concentrations. Indeed, regardless of randomization, 3HB concentrations decreased from ICU admission until day 1 to very low levels and remained suppressed until day 3 in ICU. These data suggest that the protective effects of tight glucose control in these RCTs were not mediated by increased ketone availability.

The data contrast with a previous pilot crossover RCT performed in critically ill adults, which found increased ketone concentrations by tight glucose control [15]. However, in this crossover RCT, patients received significantly less nutritional intake while receiving tight glucose control, which may have confounded the results. In the current study, nutritional intake was comparable in tight and liberal glucose control patients, as all patients received early parenteral nutrition as part of the contemporary standard of care. Interestingly, the observed suppression of ketone concentrations in both critically ill children and adults from day 1 in ICU onward mirrors the suppression observed earlier by providing early parenteral nutrition as compared with no parenteral nutrition during the first week in the ICU [9, 10]. The powerful suppression of ketogenesis by providing early full nutritional support may be difficult to counter by lowering glucose concentrations with insulin. It currently remains unclear whether tight glucose control in the absence of early parenteral nutrition affects ketogenesis, and whether this is associated with clinical benefit, which needs to be investigated [21]. Nevertheless, the current study suggests that the protective effects of tight glucose control in the context of providing early parenteral nutrition are mediated by other pathways than activation of ketogenesis. In this regard, previous studies have put forward prevention of intracellular glucose toxicity and hereby avoidance of mitochondrial damage in vital organs as potentially protective pathways activated by blood glucose lowering [22].

As in previous studies, upon ICU admission 3HB concentrations were higher in children than in adults [9, 10]. Also in health, the ketogenic response is known to be more pronounced in children [23]. Although we do not have data on pre-admission nutritional intake, most patients were presumably fasted prior to ICU admission. Hence, the significant decline in 3HB concentrations soon after ICU admission is likely explained by the initiation of early nutritional support. Alternatively, ketogenesis could be suppressed by even small doses of insulin initiated after ICU admission [23], also in liberal glucose control patients. However, in such case, one would have expected a less powerful or temporary suppression of ketogenesis in patients receiving liberal glucose control, since the administered insulin dose was significantly lower, with a considerable number of patients requiring no or only temporary insulin treatment.

This study was performed on prospectively collected samples obtained in the context of 3 large RCTs that showed benefit of tight glucose control in the ICU, which is a strength. This study inherently also has limitations. For feasibility reasons and due to missing samples, we restricted the analyses to a subset of patients at four time points. However, patients were well matched for baseline characteristics, and the outcome differences in the adult cohort mimicked the difference in the total cohort [20], suggesting the subsets are representative for the total study population. Moreover, the consistent results in both critically ill adults and children corroborate our findings. Nevertheless, we cannot exclude the possibility that the subset of patients is not fully representative for the total study population, or that the intervention may have affected ketone concentrations at other time points. Second, we cannot exclude potential bias by prolonged storage of samples. However, all samples were stored at -80 °C, and the measured range of ketone concentrations was comparable to the obtained range in more recent patient studies [9, 10]. Moreover, we previously found no impact of a freeze-thaw cycle and short-term storage at ambient temperature on 3HB measurements [24]. Hence, we consider artifacts induced by prolonged cold storage unlikely. Third, since a considerable number of samples had results for 3HB below the detection limit of the assay, we cannot exclude a minor effect of the intervention on ketogenesis. However, if present, such a small effect would likely be clinically irrelevant.

## Conclusion

Tight glucose control did not significantly affect circulating 3HB concentrations in critically ill children and adults receiving early parenteral nutrition. These data suggest that the protective effects of tight glucose control in this context could not be attributed to increased ketone body availability.

## Supplementary Information

**Additional file 1**. Table S1 Baseline characteristics and outcome of matched adults

## Data Availability

Data sharing will be considered under the form of collaborative projects. Proposals can be directed to the corresponding author.
