# Supplementary material for: Impact of tight glucose control on circulating 3-hydroxybutyrate in critically ill patients
Source: Crit Care. 2021 Oct 25;25:373. doi: 10.1186/s13054-021-03772-6 (PMC8547101; doi:10.1186/s13054-021-03772-6)
Supplement: Supplementary file 1 — Additional file 1. Table S1 Baseline characteristics and outcome of matched adults [file 13054_2021_3772_MOESM1_ESM.pdf]

Impact of tight glucose control

on circulating 3-hydroxybutyrate in critically ill patients

Additional file 1

Jan Gunst*, Astrid De Bruyn*, Michael P. Casaer,

Sarah Vander Perre, Lies Langouche, Greet Van den Berghe

* Equal contribution

Clinical Division and Laboratory of Intensive Care Medicine, Department of Cellular and Molecular Medicine, KU Leuven, Leuven, Belgium

Table S1. Baseline characteristics and outcome of matched adults

	SURGICAL ICU			MEDICAL ICU		
BASELINE CHARACTERISTICS	LIBERAL GC (N=30)	TIGHT GC (N=30)	P-VALUE	LIBERAL GC (N=20)	TIGHT GC (N=20)	P-VALUE
Age (years) - median (IQR)	64 (55-74)	65 (50-71)	1.00	66 (56-75)	68 (59-77)	0.66
Length (cm) – median (IQR)	170 (165-175)	174 (170-180)	0.11	170 (166-179)	170 (166-175)	0.80
BMI (kg/m²) – median (IQR)	25.2 (22.9-29.3)	26.2 (21.8-28.1)	0.87	22.3 (19.6-25.7)	24.5 (22.5-25.9)	0.14
Sex (male) - no. (%)	26 (87)	28 (93)	0.67	15 (75)	17 (85)	0.69
Diagnostic group – no. (%)			1.00			1.00
Surgical						
Transplantation – no. (%)	4 (13)	2 (7)				
Cardiac surgery – no. (%)	17 (57)	19 (63)				
Other surgery– no. (%)	9 (30)	9 (30)				
Medical – no. (%)				20 (100)	20 (100)	
History of malignancy – no. (%)	1 (3)	1 (3)	1.00	5 (25)	5 (25)	1.00
APACHE-II score – median (IQR)	8 (7-12)	9 (6-11)	0.76	21 (16-31)	23 (18-26)	0.98
OUTCOME						
ICU mortality – no. (%)	5 (17)	1 (3)	0.19	11 (55)	4 (20)	0.05
Length of ICU stay (days) – median (IQR)	4 (3-15)	4 (4-13)	0.94	10 (6-23)	7 (5-15)	0.09

BMI=body mass index. APACHE-II score=Acute Physiology and Chronic Health Evaluation II
